# Anti-COVID-19 Potential of Ellagic Acid and Polyphenols of *Punica granatum* L.

**DOI:** 10.3390/molecules28093772

**Published:** 2023-04-27

**Authors:** Ralitza Alexova, Simona Alexandrova, Stela Dragomanova, Reni Kalfin, Ayten Solak, Sidharth Mehan, Maria Cristina Petralia, Paolo Fagone, Katia Mangano, Ferdinando Nicoletti, Lyubka Tancheva

**Affiliations:** 1 Department of Medical Chemistry and Biochemistry, Medical Faculty, Medical University—Sofia, Zdrave Str. 2, 1431 Sofia, Bulgaria; 2 Department of Biological Effects of Natural and Synthetic Substances, Institute of Neurobiology, Bulgarian Academy of Sciences, Acad. Georgi Bonchev Str., Block 23, 1113 Sofia, Bulgaria; 3 Department of Pharmacology, Toxicology and Pharmacotherapy, Faculty of Pharmacy, Medical University, Marin Drinov Str. 55, 9002 Varna, Bulgaria; 4 Department of Healthcare, South-West University “Neofit Rilski”, Ivan Mihailov Str. 66, 2700 Blagoevgrad, Bulgaria; 5 Institute of Cryobiology and Food Technologies, Cherni Vrah Blvd. 5, 1407 Sofia, Bulgaria; 6 Department of Pharmacology, Division of Neuroscience, ISF College of Pharmacy, Moga 142001, India; 7 Department of Clinical and Experimental Medicine, University of Messina, 98122 Messina, Italy; 8 Department of Biomedical and Biotechnological Sciences, University of Catania, Via S. Sofia 89, 95123 Catania, Italy

**Keywords:** *Punica granatum*, COVID-19, polyphenols, ellagitannins, ellagic acid

## Abstract

Pomegranate (*Punica granatum* L.) is a rich source of polyphenols, including ellagitannins and ellagic acid. The plant is used in traditional medicine, and its purified components can provide anti-inflammatory and antioxidant activity and support of host defenses during viral infection and recovery from disease. Current data show that pomegranate polyphenol extract and its ellagitannin components and metabolites exert their beneficial effects by controlling immune cell infiltration, regulating the cytokine secretion and reactive oxygen and nitrogen species production, and by modulating the activity of the NFκB pathway. In vitro, pomegranate extracts and ellagitannins interact with and inhibit the infectivity of a range of viruses, including SARS-CoV-2. In silico docking studies show that ellagitannins bind to several SARS-CoV-2 and human proteins, including a number of proteases. This warrants further exploration of polyphenol–viral and polyphenol–host interactions in in vitro and in vivo studies. Pomegranate extracts, ellagitannins and ellagic acid are promising agents to target the SARS-CoV-2 virus and to restrict the host inflammatory response to viral infections, as well as to supplement the depleted host antioxidant levels during the stage of recovery from COVID-19.

## 1. Introduction

The novel severe acute respiratory syndrome coronavirus 2 (SARS-CoV-2) is a β-coronavirus with a positive-sense ssRNA genome, which is the causative agent of the coronavirus disease (COVID-19). COVID-19 presents a significant health and social burden due to the high risk of severe illness, which may require hospitalization and can lead to mortality and morbidity because of the possible long recovery periods, even after the resolution of the acute respiratory involvement stage.

In the beginning of the pandemic, the medical and scientific community was focused on developing vaccines and therapeutic approaches to manage the acute stages of the disease and to decrease mortality and the spread of the infection. However, with the establishment of effective therapeutic strategies, clinicians started to come across a condition that was later defined as post-COVID-19 syndrome or long COVID. According to the Centers for Disease Control and Prevention, post-COVID-19 is characterized by a variety of symptoms, which may last for weeks or months after the infection is resolved. Post-COVID-19 conditions affect various organ systems and may include general symptoms (fatigue, fever and post-exertion malaise), respiratory and heart symptoms, neurological symptoms and digestive symptoms, as well as other symptoms like joint pain, rash and changes in menstrual cycle (https://www.cdc.gov/coronavirus/2019-ncov/long-term-effects/index.html, accessed on 10 February 2023).

**Figure 1:**
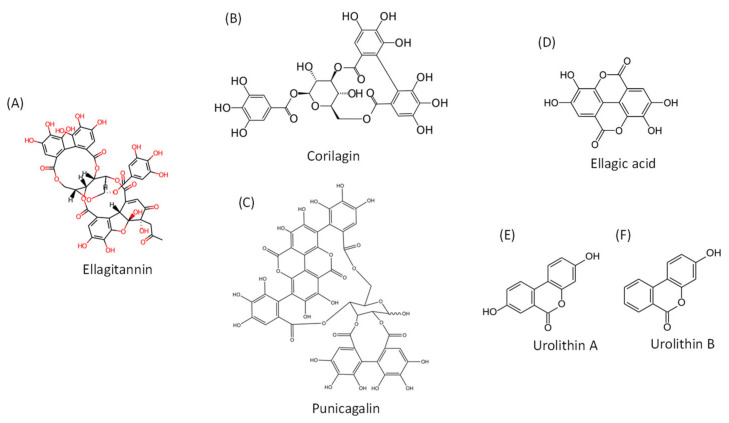
Chemical structures of ellagitannins, ellagic acid and their metabolites. (**A**). General chemical structure of ellagitannins; (**B**). Structure of corilagin; (**C**). Structure of punicagalin; (**D**). Structure of ellagic acid; (**E**). Structure of urolithin A; (**F**). Structure of urolithin B.

Recently published data suggest the use of plant polyphenolic extracts as a promising approach in the prevention of and recovery from COVID-19 [1,2,3,4,5,6]. Pomegranate (*Punica granatum*, L.) is an edible polyphenol-rich plant that has been used in traditional medicine for centuries. It is a source of several classes of polyphenols, including anthocyanins, flavonols and tannins [7]. Ellagic acid (EA) is a constituent of the hydrolysable ellagitannins (ETs) that are also present in other fruit, nuts and medicinal plants but are abundant in the pomegranate peel extracts (Figure 1). The anti-inflammatory properties of ETs and their ability to act as antioxidants and to mitigate reactive oxygen and nitrogen species (RONS) generation likely contribute to the numerous health benefits attributed to pomegranates.

Data from our previous studies, as well as the data available from other sources, support the possibility that pomegranate extract could be used as an anti-inflammatory and antioxidant agent to improve the host immune responses, including those directed against respiratory infections [8,9,10,11,12,13,14,15,16,17,18,19,20,21,22,23,24]. Some studies have reported direct interaction of EA and EA-containing polyphenols with viral components and inhibition of viral activity [10,18,25,26]. Other authors have explored the ability of pomegranate polyphenols to modulate the host immune responses to viral infection and concluded that EA and the ETs could be promising agents in preventing and ameliorating damage from COVID-19 [19,27,28,29,30,31].

## 2. COVID-19 Infection Pathology

The disease progression of COVID-19 is markedly different from that caused by the related coronavirus infections SARS-CoV-1 and MERS, even though SARS-CoV-2 infection also occurs mainly via the respiratory route, and viral entry into the host cells is facilitated by binding to the ACE2 (angiotensin-converting enzyme 2) receptor [31,32,33,34,35,36,37]. With the unfolding of the global COVID-19 pandemic, it has emerged that COVID-19 is not limited to acute infection of the respiratory tract but is a multi-organ disease with pathological changes that may last many months past the acute phase [31,32,38,39,40].

Deregulated neutrophil responses in COVID-19 have been proposed as the cause of the spreading inflammation, leading to severe systemic disease and multi-organ damage [41]. Neutrophil activation is modulated by RONS levels, which, in COVID-19, appear to result from the depletion of host antioxidant defenses [41,42,43]. The cellular effects of the SARS-CoV-1 main protease 3CL^pro^ on human promonocyte cells, such as growth arrest and apoptosis via caspase-3 and caspase-9 activities, have been well characterized. [44] RONS production and signaling through the nuclear factor kappa-light-chain-enhancer (NF-κB) pathway are likely to be associated with SARS-CoV 3CL^pro^-induced pathology and with SARS-CoV-2 pathology given the involvement of its nonstructural proteins (3CL^pro^, nsp9, nsp10) in NF-κB signaling [44,45,46].

Oxidative stress has been recognized as a pathogenetic factor in many viral infections [47,48,49]. Previous studies from our group have explored the role of oxidative stress in a model of experimental influenza viral infection induced in ICR mice [28].

Altered redox balance and enhanced production of RONS can induce cell death and the release of virions and thus represents a mode for viral dissemination. On the other hand, oxidative stress is an important trigger for the antiviral immune response [47,48,49,50,51], pointing out the importance of understanding the effects of antioxidants on humoral immunity during infection and immunization. Indeed, Crump and colleagues (2013) showed that antioxidant treatment reduces the expansion of virus-specific antibody-secreting cells through decreased proliferation and increased cell death during the effector phase [51].

If the strength of the immune response is not tightly regulated, it may lead to a cytokine storm and severe inflammation, which affect lung function, as has been observed in the progression of severe COVID-19 [39]. Such deregulated production of cytokines during the cytokine storm may be even more detrimental to lung tissues in respiratory infections than the viruses themselves. Therefore, various agents have been evaluated and are used as remedies to target not only the viruses but also the associated inflammatory immune responses [52,53].

In support of the involvement of RONS in COVID-19 pathogenesis, the levels of superoxide dismutase 3 (SOD3) in the lungs of elderly patients and children may be a factor for the different disease progression in these age groups [41,42]. It is well known that ageing is associated with a decreased capacity of the cells to maintain their redox balance and to repair subcellular components such as mitochondria through autophagy. This results in chronic low-grade inflammation, or “inflamm-ageing” [31,54,55]. Atherosclerosis, hypertension, obesity, type 2 diabetes, dementia and ageing itself are not only comorbidity factors for worse SARS-CoV-2 infection outcomes but are also emerging as diseases with significant chronic inflammation and ROS involvement [55,56,57,58]. Therefore, proactive control of pro-inflammatory conditions or the limitation of chronic inflammatory conditions may alleviate COVID-19 symptoms and aid recovery. From this point of view, antioxidant supplementation is expected to ameliorate the consequences of infection. Our studies on the subject [19,27,28,29,30] showed the positive role of antioxidant therapy in infected cells and animals, in agreement with other reports [47].

## 3. Antioxidant and Anti-Inflammatory Activity of Pomegranate Extract

The anti-inflammatory and antioxidant properties of pomegranate are attributed predominantly to the polyphenolic substances present in both the edible and non-edible parts of the plant. These polyphenols are mainly anthocyanins, condensed tannins that give the fruit its brilliant red color and hydrolysable ellagitannins (ETs) [7]. The ETs are regarded as the main contributors to the antioxidant effects of pomegranate extracts, and their concentration is much higher in pomegranate plants compared to other plants [59,60,61]. ETs consist of one or multiple units of EA attached to a sugar or a sugar alcohol core. In pomegranate extract, numerous ET compounds have been identified, the punicalagins (PUN) being the most abundant, and a smaller portion is contributed by their hydrolysis products, punicalin and free EA [22,62,63]. Purified ETs, as well as the pomegranate polyphenol extract itself, have shown good antioxidant and anti-inflammatory activity in a range of experimental systems. Numerous articles have examined their effect on chronic inflammatory conditions, including autoimmune disorders, neurodegenerative conditions, respiratory distress and viral infection. The studies show a general trend of decrease in the levels of pro-inflammatory markers after treatment with plant polyphenol-rich extracts or with their purified components and downstream metabolites [63,64,65,66,67,68,69,70]. The data show that pre-treatment with pomegranate extracts, ETs (corilagin or punicalagin) and urolithin A are associated with anti-inflammatory effects in various tissues [71,72]. 

## 4. Antioxidant and Anti-Inflammatory Effects of EA and Its Metabolites

ETs undergo hydrolysis during fruit processing or after ingestion. Therefore, ET-rich plants or plant extracts can be a nutritional source of EA. The resultant EA is further converted to urolithins by the gut flora [63,73]. The urolithins and their conjugates show higher bioavailability compared to the EA precursor and thus can be expected to exert systemic effects [74,75]. However, the human population can be divided into three different metabotypes according to the urolithin profile measured after ingestion of ET-containing foods or extracts, which may result in a high variability of the effects associated with urolithin treatment in vivo [74,75,76,77].

Chronic inflammatory conditions are associated with immune cell invasion of the tissues and often lead to tissue damage, including fibrosis. The ET corilagin and EA have been shown to be able to interfere with hypertrophic scar formation and lung fibrosis by regulating levels of TGF-β1 via activity of lysyl oxidase homolog 2 enzyme (LOXL2) and the remodeling of the extracellular matrix by matrix metalloproteinases (MMPs) [78,79]. EA supports endothelial function not only by directly reducing oxidative stress but also by decreasing the TNF-α-induced endothelial expression of vascular cell adhesion molecule 1 (VCAM1) and intracellular adhesion molecule 1 (ICAM1) [80,81]. A reduction in immune cell invasion was achieved by using pomegranate extract, PUN or urolithin A in the lungs, CNS and other inflammation sites in a variety of rodent model systems. The positive effects of ETs and related metabolites on inhibiting the invasion of CNS tissues with immune cells and the decreased activation of resident immune cells (e.g., microglia) points to the potential benefits of using plant polyphenolic extracts as part of supportive treatment for neuro-inflammation after COVID-19, a serious and long-term complication [32,38].

In addition to infiltrating the inflamed tissues, activated immune cells release pro-inflammatory cytokines (including TNF-α, IL-1β and IL-6) and pro-inflammatory molecules, such as NO, which can also influence chemotaxis. Viral infections are also able to induce the secretion of these molecules [47,50,55,65]. The SARS-CoV-2 proteins nsp9 and nsp10 may stimulate chemotaxis via IL-6 and IL-8 by interfering with NFκB signaling [46,82].

The nuclear factor NFκB has been described as a “matchmaker between inflammation, inflammatory bowel disease, cancer and diabetes” [69], and it is under its regulation that IL-6, TNF-α and IL-1β levels increase in chronic diseases. Viral infection can also be an activator for NFκB. It appears that pomegranate polyphenolic extracts and their components restrict the secretion of pro-inflammatory molecules listed above by reducing NFκB activity [21,22,23,24]. A comparative study testing three ETs (urolithin A, *iso*-urolithin A and urolithin B), along with their respective glucuronides, on lipopolysaccharide (LPS)-induced inflammation in vitro showed that urolithin A was the most effective in reducing the levels of TNF-alpha, while its glucuronide conjugate did not have any effect [71].

The ability of ETs and EA to regulate cytokine levels may be beneficial to counteract the deregulation of immunity induced by SARS-CoV-2.

The studies demonstrating the antioxidant and anti-inflammatory properties of plant extracts containing ellagitannins or of purified ellagitannins and downstream metabolites (ellagic acid or urolithins) are listed in Table 1.

## 5. Antiviral Activity of Pomegranate Polyphenolic Extracts and Ellagic Acid

Pomegranate extracts from different parts of the plant, purified ETs of pomegranate origin or from other medicinal plants, and EA have been tested and have shown broad antiviral activity [20,21,22,23,24,25,26,91,92,93,94,95,96,97,98,99], as summarized in Table 2. The direct application of EA-containing polyphenol extracts to tissue-cultured cells showed good antiviral activity, including for some models of viral respiratory tract infection, and was accompanied by low cytotoxicity. An IC_50_ value lower than 0.06 mg/mL was obtained for the inhibitory activity of pomegranate peel extract to the interaction of the S1 subunit of the SARS-CoV-2 spike protein with host ACE2, compared to its constituent punicalin (IC_50_ of 0.06 mg/mL), suggesting synergism with other components in the plant extract [91].

Pomegranate leaf ethanolic extract showed antiviral activity against Zika virus and *herpes simplex* virus type 2 (HSV-2) [26], while a pomegranate phenolic extract showed inhibitory activity against influenza [22], and extracts from the fruit (juice and peel) were active against hepatitis C virus (HCV) and SARS-CoV-2 [26,91]. Similarly, promising results against a range of viruses were obtained with purified components of these extracts, the dominant pomegranate ET punicalagin/punicalin fraction and the ET hydrolysis product EA (Table 2). Chebulagic acid, another ET from the Japanese medicinal plant *Geranium thunbergii,* also exerts broad antiviral activity with effects similar to punicalagin [100,101,102]. These ETs both seemingly interact with viral glycoproteins and glycosaminoglycan molecules on the host cell surface, which assist the entry into host cells for a range of viruses [25,93].

The antiviral effect of purified EA against viruses such as Zika, HRV-2, HRV-3, HRV-4 and influenza has been suggested to occur by disrupting the virus’s interaction with the host cell surface [22,23,24]. EA may also be the dominant antiviral substance in pomegranate leaf extract, according to Acquadro et al. [24], as punicalagins and punicalins are not present in detectable concentrations in the leaves of the plant.

The effects of EA on the virus may extend to other mechanisms, as in human immunodeficiency virus-1 (HIV-1) infection, this phytochemical restricted viral replication by inhibition of the viral integrase, but not protease [98]. In hepatitis B virus (HBV) infection, on the other hand, EA restricted viral proliferation by preventing hepatitis Be antigen (HBeAg) secretion [99].

**Table 2:** Antiviral properties of plant extracts containing ellagitannins or of purified ellagitannins and downstream metabolites (ellagic acid or urolithins). DENV: Dengue virus; HBV: Hepatitis B virus; HCMV: Human cytomegalovirus; HCV: Hepatitis C virus; HIV: Human immunodeficiency virus; HRV: Human rhinovirus; HSV: Herpes simplex virus; MV: Measles virus; RSV: Respiratory syncytial virus.

Compound Tested	Viral Target	Molecular Mechanism	References
ellagic acid	influenza A	synergistic effect on antioxidant defenses with oseltamivir and isoprinosine	[21]
pomegranate polyphenol extract, punicalagin	influenza A influenza B	synergistic effect on viral proliferation inhibition with oseltamivir	[22]
pomegranate leaf ethanolic extract	HSV-2 Zika	reduces viral proliferation in cells	[24]
pomegranate peel extract and fruit juice	HCV	inhibition of NS3/4A protease activity	[26]
pomegranate peel extract, punicalin	SARS-CoV-2	binds to SARS-CoV-2 S-glycoprotein and inhibits binding to ACE2	[91]
*Rhodiola rosea* extract	Ebola	inhibits viral entry in cells	[20]
punicalagin and Zn(II)	SARS-CoV-2	inhibition of 3CL protease, synergistic effect with Zn(II)	[92]
chebulagic acid, punicalagin	SARS-CoV-2	non-competitive inhibition of 3CL protease	[93]
	HSV-1	inhibits viral entry in cells and cell-to-cell spread via viral glycoprotein and host glucosaminoglycans interaction	[25]
	HCMV HCV DENV MV RSV	inhibits viral attachment to cells	[94]
geraniin	SARS-CoV-2	binds SARS-CoV-2 S-glycoprotein receptor binding domain	[95]
corilagin	SARS-CoV-2	binds to SARS-CoV-2 S-glycoprotein and inhibits binding to ACE2	[96]
	SARS-CoV-2	inhibits activity of RNA-dependent RNA polymerase nsp12	[97]
ellagic acid	Zika	hypothetical interaction with cell surface to prevent viral infection	[24]
	HIV-1	blocks viral integrase but not protease	[98]
	HRV2 HRV3	reduces viral proliferation in cells	[23]
	HBV	blocks HBeAg secretion from cells	[99]
	Ebola	inhibits viral entry in cells	[20]

## 6. Binding of Ellagitannins and Ellagic Acid to Components of SARS-CoV-2 and Human Host

In the beginning of the pandemic, the availability of the SARS-CoV-2 genomic information and the crystal structure of several of its proteins and of the human ACE2 receptor allowed for in silico docking studies and screens of phytochemical libraries for appropriate inhibitor molecules. The search identified numerous plant-derived secondary metabolites, among which EA and EA-containing polyphenols were high scoring. The interactions of ETs appear to be mediated primarily by the formation of multiple H-bonds to the amino acids of the viral target protein [25,91,93,96,97,103].

The target proteins in these in silico studies include the non-structural proteins (nsp) of the virus, as well as the spike protein. As our understanding of the SARS-CoV-2 mode of infection and the active sites of the proteins involved has progressed, the predicted docking studies have continued to grow in number and have also been expanded to include human target proteins. Some of these have also been tested in vitro to confirm the computational predictions in binding assays on purified proteins, and the dose-dependent antiviral effect of the purified polyphenols as well as whole pomegranate peel extract against SARS-CoV-2 was supported [92,93,95,96,104].

The receptor binding domain (RBD) in the N’ domain (NTD) of the spike (S) protein of SARS-CoV-2 recognizes the protease site of ACE2 on the surface of human cells. The S and M proteins also gather the viral components during intracellular replication. Thus, the RBD of the S protein has been a target of high interest in the search for plant metabolites with potential antiviral activity. ETs including PUN, corilagin, geraniin, punicalin and EA were identified as binding this viral protein [105]. The interaction was confirmed by an in vitro assay, which showed that punicalin interfered with the S1–ACE2 interaction [88]. Interestingly, in some but not all docking studies, the glycosylated N343 residue of the S1 RBD was identified as interacting with ETs [91,96,105,106].

The SARS-CoV main protease Mpro (3-chymotrypsin-like protease 3CL^pro^ or Nsp5) is a Cys protease that cleaves the viral polyproteins at 11 different sites and is thus a good target for interfering with viral replication [107]. The cleavage site recognized by the protease appears in proteins involved in human innate immunity and may interfere with the immune response to viral infection [45]. Due to the apparent suitability of 3CL^pro^ for drug development, a vast range of studies have included plant polyphenol-3CL^pro^ docking, including PUN, punicalin, corilagin, chebulagic acid and geraniin, as well as EA and urolithins [93,104,106,107,108,109,110,111,112,113,114]. The catalytic dyad of 3CL^pro^ is composed of H41 and C145, and in silico docking showed that EA and its precursors form H-bonds with the catalytic C145 residue [104,106,107,108,114]. Residues in the protease, which also showed up as interacting in many studies, were the nearby position G143 as well as E166 and Q189, all of which comprise part of the substrate-binding site of the protease [45]. On the other hand, in vitro studies by Du et al. (2021) indicated noncompetitive binding of ellagitannins to the viral protease and therefore searched for an allosteric site in the protein molecule, which they identified as a cleft between domains II and III [93].

Bahun and coworkers [104] followed up on their in silico docking studies with in vitro protease inhibition and surface plasmon resonance and calculated a K_d_ of 311 ± 69 µM for the EA-3CL^pro^ interaction and an IC_50_ of 11.8 μM. Surface plasmon resonance also revealed strong binding to the main protease, while EA had lower affinity to the enzyme, and the urolithins were weaker inhibitors [113]. In another study, the IC_50_ of PUN towards 3CL^pro^ 5.7 µM was enhanced in the presence of Zn^2+^ [92]. Corilagin scored in the top eight molecules in an in vitro fluorescent assay for protease inhibition [115]. A similar assay calculated IC_50_ 9.09 ± 0.87 μM for chebulagic acid and 4.62 ± 0.27 μM for punicalagin [93]. Also, an in vitro study comparing the inhibitory activity of PUN, EA and gallic acid, as well as whole pomegranate peel extract, found 80% inhibition of 3CL^pro^ activity when the pomegranate extract was used at 0.2 mg/mL, and most of this effect was contributed by PUN [116].

In addition to the intensively explored interaction of ETs with 3CL^pro^, ETs also appear to bind to and interfere with the functioning of some of the remaining 15 non-structural proteins of SARS-CoV-2. EA was the best in silico candidate for nsp9 binding and also showed nsp10 binding energies similar to other tested bioactive components from *Moringa oleifera* [103]. The two viral proteins nsp9 and nsp10 (methyltransferase) interact with NFκB repressing factor (NKRF) and may interfere with NFκB transcription activity, for which NKRF competes [46]. Nsp15 (endonuclease) and PUN were identified as interacting in silico [110]. A more extensive screen identified punicalin as the lowest binding energy partner for RdRp (nsp12, RNA-dependent RNA polymerase), as well as for nsp9 and PL^pro^ (nsp3, papain-like protease) [1]. In a biosensor-based assay, corilagin was also the best binding partner for RdRp and at 40 μM inhibited the polymerase activity by more than 80% [97]. In a cell-based assay, this activity of corilagin persisted and inhibited infection of Vero cells by HCoV-OC-43 and SARS-CoV-2, indicating a broader coronavirus recognition [97].

The virus also interacts with several host proteases, including transmembrane serine protease 2 (TMPRSS2), furin, cathepsin L and ACE2. These were also tested for interaction with ETs and show additional potential of ETs as host protease inhibitors.

ACE2 acts as a metallocarboxypeptidase that is distant from the ACE2 catalytic site. The ellagitannins PUN, punicalin and EA recognized this human enzyme [105].

Pedunculagin, punicalin, PUN and EA bound in silico to furin at overlapping regions that included the catalytic residues H194, N295 and S368 [105,117]. Furin is a subtilisin-like serine endopeptidase that facilitates the entry of several viruses, including Ebola, HIV, Dengue and influenza, into host cells and activates the spike protein of SARS-CoV-2 by cleaving the spike glycoprotein at the S1 site [117].

Transmembrane protease serine 2 is a membrane-bound enzyme needed for viral entry. It exposes the viral fusion machinery by performing a cleavage on the S protein secondary to furin at the S2’ site and potentially the T1 and T2 sites [118,119]. This protease also facilitates the fusion of influenza A and B viruses with the host cell membrane [118]. The proteolytic processing of the spike protein also releases S1, which can act as an immune decoy [118]. The active site amino acids in TMPRSS2 (Q276, E299, K300, P301, K340, K342, E389, K390, L419, S441, Q438, and W461) and the catalytic triad H296, D345 and S441 interact with the SARS-CoV2 S-protein. They also appear to be recognized by pedunculagin and PUN and by bromhexine and serine protease inhibitors, such as camostat, as agents that inhibit TMPRSS2 and have been considered in COVID-19 treatment [110,117,119]. Camostat and nafamostat act by acetylating the S441 residue, which is also recognized by the ETs [118]. A combination of PUN with 2-deoxy-d-glucose showed the strongest binding energy (−10.8 kcal/mol) to TMPRSS2, more favorable than the established inhibitors bromhexine and camostat (approximately −9 kcal/mol) [110,119]. On the other hand, Surucic et al. [105] identified a different site of TMPRSS2 as the target for camostat, EA and ETs binding (including R87, R91, M97, R405, M404), with lower calculated binding energies. While this may be a potential allosteric site, the authors did not consider the intramembrane region (amino acids 1–145) of TMPRSS2, which is unlikely to be accessible by ellagitannins when the protease is in the plasma membrane [119]. A screen of several thousand plant secondary metabolites identified ellagitannins, including punicalin, pedunculagin and granatin A and B as compounds with good binding energy to TMPRSS2 and an ET metabolite 3′-*O*-methyl ellagic acid 4-xyloside with somewhat lower binding energy [5].

## 7. Considerations for the In Vivo Administration of EA-Containing Extracts

The low bioavailability of both EA and ETs after oral ingestion and their extensive biotransformation in the digestive tract makes it unlikely that their antiviral activity in vivo would be directly comparable to what is observed in cultured cells. In vivo exposure to highly concentrated ETs, resembling the conditions used in the studies listed in Table 2, will probably not go beyond the upper respiratory tract. A feasible application method may be lozenges, a nasal spray or microencapsulated pomegranate polyphenolic extract, such as the one used with good results in a murine model of asthma [61,120,121]. EA and the precursor ETs have low bioavailability if ingested but have high potential of being strong antioxidants and anti-inflammatory compounds and may be valuable in inhibiting viral particles if applied directly in the upper respiratory tract.

Nevertheless, the broad antiviral, antioxidant and anti-inflammatory effects of dietary intake of pomegranate extracts cannot be fully explained by direct polyphenols–virus interaction. As evidenced by the comparison of the data in Table 1 and Table 2, the numerous benefits of EA and of the more bioavailable urolithins are probably due to the convergence of poorly understood mechanisms that affect the bioactivity of ETs, resulting in immune cell regulation and signaling. We can also expect that the overall antioxidant and anti-inflammatory properties of ETs may also provide benefits in recovery from long COVID [19,27,28,29,30,34,81].

## 8. Conclusions

In silico and in vitro studies show that EA and ETs bind to several components of the SARS-CoV-2 virus with different affinity. This complementary effect may explain the higher antiviral activity of whole pomegranate extract relative to its isolated components. Other flavonoids also interact with the same SARS-CoV-2 targets and are present in ET-containing plants. These synergistic effects deserve further exploration in vivo. While the SARS-CoV-2 virus can lead to acute and prolonged disease by deregulating host immunity, triggering systemic inflammatory responses, and depleting intrinsic antioxidants, the EA-containing polyphenols may be promising candidates in countering all the above. Pomegranate extracts, containing a multitude of EA-derivatives, appear to act through different mechanisms to maintain endothelia integrity, to restrict inflammatory cell activation and tissue invasion and to supplement the antioxidant systems in the body. Supplementation with these bioactive compounds may therefore be especially beneficial to individuals with high risk for severe COVID-19 progression, e.g., ageing, type 2 diabetes, cardiovascular pathology, atherosclerosis and neurodegenerative diseases.

## Figures and Tables

**Table 1:** Antioxidant and anti-inflammatory properties of in vitro and in vivo application of plant extracts containing ellagitannins or application of purified ellagitannins and downstream metabolites (ellagic acid or urolithins)*. ↑*: increased; ↓: decreased; x: counteracted.

Compound Tested	Experimental System	Findings	References
pomegranate extract	human consumption of capsules	↑ antioxidant capacity of plasma (ORAC) within 30 min	[83]
	Alzheimer’s disease transgenic R1.40 mice model	non-significant ↓ TNFα, IL-1 and COX2	[84]
pomegranate flower extract	Zucker diabetic fatty rat	↓ interstitial and perivascular collagen accumulation in heart, expression of collagen I, collagen III, fibronectin, ET1, ETA, ETB, x NFκB activity	[85]
pomegranate juice	hyperoxia rat model	↓ neutrophil infiltration, albumin leak, ROS, apoptotic bodies in lungs, IL-1β, IL-6	[86]
pomegranate leaf ethanolic extract	intranasal application in asthma mouse model	↓ IL-1β, IL-5, inflammatory cell infiltration in lung, mucous glycoprotein secretion	[64]
pomegranate peel extract	neutrophil culture and LPS-stimulated mice	x MPO activity in neutrophils, ↓ lung invasion of inflammatory cells	[87]
	LPS-induced RAW264.7 macrophages	↓ TLR4 expression, ↓ IL-1β, IL-6, TNFα, NO, PGE2, ROS production, x nuclear translocation of NFκB nuclear translocation	[70]
walnut methanolic extract	human aorta endothelial cells (HAEC)	↓ TNFα-induced VCAM1 and ICAM1 expression	[80]
	KS483 osteoblastic cells line	nodule formation induced	
corilagin	HSV-1 infected MV-2 microglia cells	↓ secretion of NO, TNFα, IL-1β, ↑ secretion of IL-10, cytochrome c, caspase-3, -8, -9 and -12	[65]
	HSV-1 infected mice	↓ numbers of inflammatory cells in the brain, ↓ neuronal degeneration and interstitial edema	
punicalagin	acute respiratory distress mouse model	↓ inflammatory cell lung invasion, alveolar wall thickening, pulmonary congestion, ↓ TNFα, IL-1β, and IL-6 levels, MPO activity, TLR4 expression, x phosphorylation of IκBα and NFκB p65	[67]
	Jurkat cells	T cell activation by NFAT	[88]
	activated CD4+ murine splenic lymphocytes	↓ IL-2 mRNA and protein	
	PMA-induced ear edema in mice	↓ hyperplasia and inflammatory cell infiltration	
	LPS-induced RAW264.7 macrophages	↓ TLR4 expression, ↓ IL-1β, IL-6, TNFα, NO, PGE2, ROS production, x nuclear translocation of NFκB nuclear translocation	[70]
ellagic acid	human aorta endothelial cells (HAEC)	↓ TNFα-induced VCAM1 and ICAM1 expression	[80]
	KS483 osteoblastic cells line	nodule formation induced	
	mice on high fat diet	↓ aortic lesions, plasma cholesterol and triglyceride, ↓sICAM1 and E-selectin expression, ↑ Nrf2, HO-1 protein and aortic NOS activity	[81]
	human umbilical vein endothelial cells (HUVEC)	Nrf2-mediated cytoprotection, ↑ HO-1 protein	
	human Caco-2 intestinal cells	↓ NFκB activation after LPS stimulation, ↑ IκB-α phosphorylation and IL-8 secretion after IL-1β stimulation	[89]
	in combination with oseltamivir and isoprinosine in influenza A infected mice	↑ glutathione reductase activity, ↓ TBARS in blood plasma and lungs during infection	[21]
	LPS-induced RAW264.7 macrophages	↓ TLR4 expression, ↓ IL-1β, IL-6, TNFα, NO, PGE2, ROS production, x nuclear translocation of NFκB nuclear translocation	[70]
	Caco-2 and HT-29/B6 intestinal cells	↑ transepithelial resistance, ↓ caludin-4, -7, -15 expression	[90]
urolithin A	experimental autoimmune encephalomyelitis	↓ demyelination and inflammatory infiltrating cells, reduce severity of disease, ↓ activation of dendritic cells and CNS microglia	[68]
	bone marrow-derived dendritic cells and SIM-A9 microglia	↓ IL-1β, IL-6, TNFα, ↑ IL-10	
	inflammatory bowel disease model LPS-stimulated BMDM	↓IκB-α phosphorylation, IL-1β, IL-2, IL-6, IL-12, TNFα, NOS2, double-stranded DNA breaks, superoxide production, MAPK and PI3K activation, proinflammatory miRNAs	[69]
	Caco-2 and HT-29/B6 intestinal cells	x TNF-α induced drop in transepithelial resistance	[31]
urolithins	LPS-stimulated BV2 microglia	↓ NO, TNFα and IL-6, improved SH-SY5Y neuronal cell viability in H_2_O_2_	[63]

## Data Availability

Not applicable.
